# How Effective Is Vitamin C for Gingival Depigmentation? A Scoping Review

**DOI:** 10.1002/cre2.70272

**Published:** 2026-03-11

**Authors:** Shubhankar Mehrotra, Malvika Shyamkumar, Shravya Marcherla, Aditi Chopra, Marwa Madi

**Affiliations:** ^1^ Department of Periodontology Manipal College of Dental Sciences, Manipal, Manipal Academy of Higher Education Manipal Karnataka India; ^2^ Department of Preventive Dental Sciences, College of Dentistry Imam Abdulrahman Bin Faisal University Dammam Saudi Arabia

**Keywords:** aesthetics, gingiva, gingival depigmentation, melanin, pigmentation, Vitamin C

## Abstract

**Objectives:**

Excessive gingival pigmentation can be treated by surgically removing the pigmented gingival tissue with a scalpel, bur, LASER, electrocautery, cryotherapy, chemical peeling, or masking with free gingival grafts. Recently, vitamin C (ascorbic acid), in gel, powder, or liquid has been used alone or as an adjunct to micro‐needling or scalpel for gingival depigmentation. This paper aims to critically appraise current evidence on the role of vitamin C in gingival depigmentation.

**Material and Methods:**

The review was conducted according to the Preferred Reporting Items for Systematic Review and Meta‐analysis for Scoping Review (PRISMA‐Scoping Review). Studies using “Vitamin C” in any form, via any application method, frequency, duration, and dosage for gingival depigmentation and compared were included.

**Results:**

Out of 119, 103 articles were excluded and 16 articles were taken up for full text screening. Of 16, 12 articles were included in the review. The results showed that vitamin C has been used along with scalpel depigmentation or as a stand‐alone therapy (as mesotherapy or topical application). Vitamin C effectively reduced gingival pigmentation, however with no statistical difference between the scalpel and vitamin C application was noted. No studies have compared the efficacy of vitamin C to cryotherapy. Microneedling of gingiva followed by vitamin C application was also done. The recurrence rate of pigmentation following vitamin C mesotherapy application was 32.59% after 3 months compared to 32.87% with the scalpel. Vitamin C mesotherapy also revealed a significant reduction in VAS scores for itching and pain and better patient acceptance than scalpel depigmentation.

**Conclusion:**

Vitamin C application with microneedle or mesotherapy is a promising atraumatic alternative to a scalpel for gingival depigmentation.

## 1 Introduction

Gingiva is physiologically pigmented due to the deposition of melanin pigments by the melanocytes in the gingival tissues (Dummett 1946). However, increased melanin pigmentation may occur in some individuals due to certain local and systemic conditions and syndromes such as smoking, use of certain medications, unintended implantation of amalgam restoration, Addison's disease, Albright's syndrome, Acromegaly, etc. (Rotbeh et al. 2022; Alhajj and Alhajj 2020). According to a recent meta‐analysis, 20.8% of the population suffers from physiologic/pathologic oral hyperpigmentation, that corresponds to one out of five people. Men were more likely to be affected by the increased pigmentation than women (Rotbeh et al. 2022). Smokers or those exposed to second‐hand smoke are also more likely to be affected. Those drinking hot drinks, and dark‐skinned individuals show an increased prevalence of gingival pigmentation (Alhajj and Alhajj 2020; Scully and Bagan 2004). Approximately 30%–98% of Asians are estimated to suffer from hyperpigmented gingiva with diffuse deep purplish discoloration or irregularly shaped patches, striae, or strands (Javali et al. 2011).

Patients often seek depigmentation procedures as a result of excessive gingival pigmentation, which compromises the aesthetics. There are various methods available for removing pigmented gingival tissue: surgically through scalpel de‐epithelialization, bur abrasion, laser depigmentation, electrosurgical subtraction, cryotherapy, chemical peeling, and masking with free gingival grafts or acellular dermal matrixes (Parwani and Parwani 2013; Alhabashneh et al. 2018; Suragimath et al. 2016). Compared to all these techniques, soft tissue lasers have become increasingly popular due to improved patient satisfaction, aesthetic outcomes, shorter operating times, and decreased bleeding (Muruppel et al. 2020; Jagannathan et al. 2020). Carbon dioxide, Erbium‐doped yttrium‐aluminum‐garnet (Er: YAG), neodymium‐doped yttrium aluminum garnet (Nd: YAG), erbium, chromium: yttrium‐scandium‐gallium‐garnet (Er, Cr: YSGG), and diode are some of common lasers used for treating gingival depigmentation. Among these, diode lasers tend to have the longest time for reappearance of pigmentation, especially in non‐smoker patients (Altayeb et al. 2021).

Recently, Gul et al. conducted a systematic review comparing the efficacy of various treatment modalities to treat gingival pigmentation. They concluded that laser‐assisted depigmentation is equal to, or even better than scalpel depigmentation regarding pigmentation outcomes and recurrence. The precise mechanism of repigmentation is not yet fully understood. However, the migration theory suggests that active melanocytes from the adjacent pigmented tissues may migrate to the treated areas, potentially leading to a failure in the process. A study by Ginwalla et al. (1966) found that 50% of patients showed repigmentation after depigmentation with bur abrasion technique within 24 to 56 days. Pal et al. (1994) also noted repigmentation in 19% of patients following gingival depigmentation by surgical bur. Nevertheless, lasers have shown superior results in terms of patient acceptance, pain relief, and satisfaction when compared with surgical blades (Gul et al. 2019). A recent systematic review by Ahmed et al. (2023) including eight studies reported superior characteristics and treatment outcomes for diode lasers as compared to Erbium lasers. The time before pigmentation recurrence was longer for the diode laser compared to the Erbium laser (Ahmed et al. 2023). Inchingolo et al. (2024) also conducted a systematic review and evaluated the efficacy of the diode laser for the treatment of gingival pigmentation compared to the conventional scalpel technique and found that the diode laser provides better results in terms of intraoperative bleeding and perception of pain for the patient. However, there were no differences in depigmentation and wound healing intensity (Jnaid Harb et al. 2021). Additionally, studies have shown that diode lasers have a lower recurrence rate, but this finding has not yet been confirmed by long‐term clinical evidence (Muharib and Almasoud 2020; Alasmari 2018). Furthermore, several studies have demonstrated that both laser electrocautery and scalpels are associated with being technically demanding, time‐consuming, and increasing anxiety and discomfort in patients (Patil et al. 2015; Kumar et al. 2013; Bergamaschi et al. 1993; Kathariya and Pradeep 2011; Bakutra et al. 2017). Laser treatment has been reported to delay wound healing when compared to scalpel‐based depigmentation, especially at sites with thin gingival biotypes and narrow inter‐papillary spaces (Kathariya and Pradeep 2011 Bakutra et al. 2017). As a result, many authors promote the use of cryogens for gingival depigmentation, however, clinically, it is difficult to control the depth of penetration of cryogens into the gingival tissues, which limits their use. When applied, several cryogens have been shown to cause tissue damage and stinging or burning sensations (Kumar et al. 2013; Bergamaschi et al. 1993; Kathariya and Pradeep 2011). Accordingly, the need for exploring non‐invasive alternatives to gingival hyperpigmentation is often perceived.

**Figure 1:**
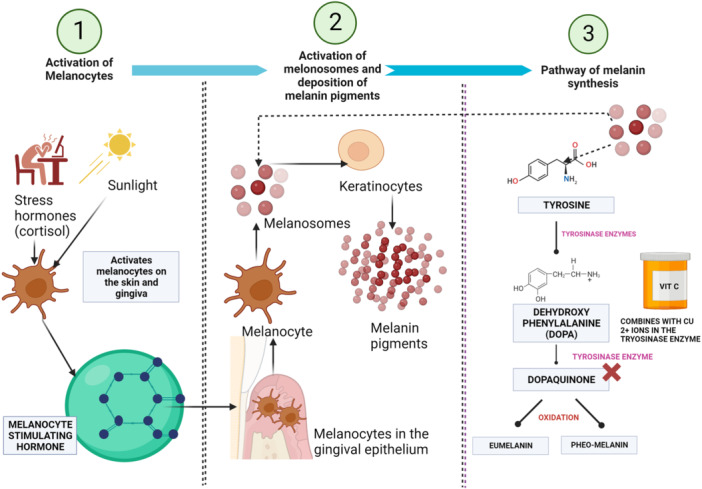
Schematic representation of explaining how Vitamin C can be used for gingival depigmentation: (1) Continuous exposure to sunlight and high stress level (increase cortisol) activates melanocytes in the gingival tissues and increase the levels of melanocyte‐stimulating hormone. (2) Melanocytes stimulating hormone stimulate melanocytes to form melanosomes, which in turn form melanin pigment. (3) The process of melanin formation takes place in the melanosomes. The tyrosine kinase enzymes in the melanosome convert dehroxyphenyalanine (DOPA) into dopaquinone. Dopaquinone gets oxidized into Eumelanin and Pheomelanin. Vitamin C can bind to the Copper ions (Cu2+) in the tyrosine enzyme and inhibit the conversion of DOPA to dopaquinone. This inhibits the process of melanin formation.

There is an emerging use of vitamin C for gingival depigmentation and aesthetic procedures (Sanadi and Deshmukh 2020; Mostafa and Alotaibi 2022; Sheel et al. 2015; Shimada et al. 2009; Lerner and Fitzpatrick 1950; Yussif et al. 2017). Topical application of vitamin C in gel, powder, or liquid forms, either alone or as an adjunct to micro‐needling or scalpel, over the pigmented gingival epithelium is being used (Mostafa and Alotaibi 2022; Sheel et al. 2015; Shimada et al. 2009). Vitamin C inhibits the activity of the tyrosinase enzyme by interacting with the Copper (Cu) ions at the tyrosinase active site. This in turn reduces the melanin formation, which is responsible for converting the amino acid dehydroyphylalanine (DOPA) to dopaquinone. The inhibition of dopaquinone prevents the formation of melanin pigments (Lerner and Fitzpatrick 1950; Yussif et al. 2017) (Figure 1). Vitamin C also has an epigenetic suppressive action on cells and controls melanocyte activity (Gustafson et al. 2015). Vitamin C can even affect the intercellular junctions between melanocytes and keratinocytes and affect the HMB‐45 receptor which controls the activity of melanocytes in the suprabasal layers of the epithelium (Yussif et al. 2017). Although vitamin C has shown promising results for the depigmentation of the skin, few clinical studies have been conducted to explore its role in gingival depigmentation. To our knowledge, critically appraisal of the existing evidence on the role of vitamin C as an adjunct for gingival depigmentation is limited. With the use of vitamin C as a new method of gingival depigmentation gaining popularity, many professionals are unaware of the various types, modes of application, and current evidence on its effectiveness and long‐term prognosis. Hence, this scoping review aims to critically appraise the existing literature and provide a comprehensive discussion on the current methods and efficacy of vitamin C for gingival depigmentation.

## 2 Methodology

The review is based on the guidelines for the scoping review by the Preferred Reporting Items for Systematic Review and Meta‐analysis for Scoping Review (PRISMA‐Scoping Review) checklist (Tricco et al. 2018). The objectives of the review were:

1. To assess the current evidence regarding the efficacy and mechanism of action of vitamin C in gingival depigmentation.
2. To determine the most effective method of applying vitamin C for reducing gingival pigmentation.
3. To review the evidence regarding patient‐reported outcome measures, adverse events, and recurrence rate following vitamin C depigmentation compared to other treatment methods for gingival depigmentation.

The eligibility criteria for studies are described using the Participant‐Intervention‐Comparison‐Outcome‐Study Design framework as follows:

Types of participants (P): Participants over the age of 18 (males and females) who have increased gingival pigmentation due to heavy melanin deposition. Studies on hospitalized patients, physically or mentally handicapped patients, and patients with terminal or serious illnesses were excluded.

Intervention (I): Studies where vitamin C was used in any form, via any application method, frequency, duration, and dosage for gingival depigmentation.

Comparator (C): Studies where gingival depigmentation was done by any of the following: scalpel, laser, electrocautery, cryogen, bur abrasion, or microneedling.

Outcomes (O): Studies assessing the effect of vitamin C on the reduction in intensity, distribution, and area of pigmented gingiva; patients’ perception of aesthetics, nature of wound healing, and postoperative complications (Table 2).

Types of studies (S): Observational, case‐control, cohort, clinical trials, qualitative studies utilizing questionnaires and surveys to assess patient‐related outcomes were included. All preclinical, in‐vitro, animal, case reports, and case series studies were also included. However, all editorials, book reviews, literature reviews, and letters to the editor were excluded from this review.

### 2.1 Search Strategy, Information Sources, and Keywords

The following electronic databases: MEDLINE (PubMed), Scopus, EBSCO (dentistry and open science access), Cochrane database, Web of Sciences, and ClinicalTrial.org. were searched on 14th December 2022, and updated on February 2024. The following keywords and MeSH terms were utilized for data collection: (Vitamin C OR Ascorbic acid) AND (gingival OR gingiva OR keratinized gingiva OR pigmented gingiva) AND (melanin OR pigment* OR depigmentation). The PubMed search string was adapted to the other database. Articles written in any language were included. The search results were transferred into the “Mendeley reference manager (version 1.19.4)” and duplicates were removed. The comparator group was not added in the search string as it would exclude studies where only Vitamin C was used without any other intervention. The details of the search strings in the individual database are provided in Table 1.

**Table 1:** Search strategy for different databases for including articles for the title and abstract screening.

S/No	Database	Search string used	No of articles
	PubMed	(Vitamin C OR Ascorbic acid) AND (gingival OR gingiva OR keratinized gingiva OR pigmented gingiva) AND (melanin OR pigment* OR depigmentation)	14
	Scopus	(Vitamin C OR Ascorbic acid) AND (gingival OR gingiva OR keratinized gingiva OR pigmented gingiva) AND (melanin OR pigment* OR depigmentation)	6
	Embase	(Vitamin C OR Ascorbic acid) AND (gingival OR gingiva OR keratinized gingiva OR pigmented gingiva) AND (melanin OR pigment* OR depigmentation)	14
	Web of Science	(vitamin C OR ascorbic acid) AND (gingival OR gingiva OR keratinized gingiva OR pigmented gingiva OR melanin OR pigment OR depigmentation))	77
	Cochrane Database (clinical trials)	(Vitamin C OR Ascorbic acid) AND (gingival OR gingiva OR keratinized gingiva OR pigmented gingiva) AND (melanin OR pigment* OR depigmentation)	7
	Clinicaltrials. gov	https://clinicaltrials.gov/ct2/show/NCT03719274?cond=%28Vitamin+C+OR+Ascorbic+acid%29+AND+%28gingival+OR+gingiva+OR+keratinized+gingiva+OR+pigmented+gingiva%29+AND+%28melanin+OR+pigment*+OR+depigmentation%29&draw=2&rank=1	1
		Total search	119

### 2.2 Data Charting and Selection of Studies Process

The results from the data search were transferred into the Mendeley reference manager (version 1.19.4) and the duplicates were removed. After removing the duplicates, all articles were copied into the Microsoft Excel spreadsheet for screening. During the review process, authors from both teams (S.M. and S.H.) and (M.S. and A.C.) screened titles and abstracts for eligibility. All articles where Vitamin C was either used alone or compared to any other intervention for depigmentation of gingival tissues were included. The following articles were excluded: Articles where Vitamin C was used for any periodontal surgical procedure other than depigmentation of gingival tissues; articles where vitamin C was used as a supplement or given systematically to treat vitamin C deficiency or for treating any periodontal disease; articles where vitamin C was used for removal of pigmented tissues in any other part of the body except gingival tissues. Any discrepancies during the initial screening were resolved by involving the other reviewer (M.M.), who was not involved in the initial screening phase. Following the selection of titles and abstracts by both teams, full‐text screening was conducted. The full‐text screening was conducted by two independent reviewers (S.M. and S.M.) following the eligibility criteria. At the full‐text stage, discrepancies were resolved through mutual discussion and consultation with another reviewer (A.C.). Citations of the final included articles were copied into another Microsoft Excel 2013 version, and data were extracted independently by all authors.

### 2.3 Data Items and Synthesis of Results

The following data items were extracted from each study: Bibliographic and study details, that is, author details, funding, organization, aims and objectives of the study, type of study; participants’ detail, that is, number, age, gender, socioeconomic status, income, study settings (community/hospital/university/private clinic); contextual information; exposure details, type and mode of application of vitamin C used in the study along with the outcome details of each study. A preliminary data extraction form for all the data extracted from each study is given in Table 2. In case of missing data, the reviewers tried to obtain it by contacting the corresponding authors.

**Table 2:** Preliminary data extraction/coding form.

S/No.	Sections	Required information
1	Bibliographic details	First author, corresponding author email; year of publication; funding information Language of the paper; site of study (country)
2	Study details	Aim and objectives of the study Methodology of the study (study design, sampling, sample size [in each group if applicable], type of analysis, duration of the study; the follow‐up, drop‐outs); blinding method; allocation concealment
4	Population characteristics	Age (mean + SD) or age range; gender (male to female); nature of study population (healthy or diseased population; type of gingival pigmentation; presence or absence of any other disease reported in the patient (gingivitis/periodontitis/others).
5	Exposure details	Nature of Vitamin C (gel/liquid/powder); type or form of Vitamin C (name of vitamins C analog), dosage (mg or %), frequency (once or twice), mode of application of Vitamin C (topical/injection); ease of use; duration of surgery (mins)
6.	Comparator group	Gingival depigmentation using a scalpel, laser, electrocautery; cryogen.
7	Outcome	Gingival pigmentation score before and after the procedure, as measured by any method or index (Dummet‐Gupta Oral Pigmentation Index [DOPI], Hanioka's Melanin Index, Spectrophotometric analysis, inhibition of tyrosinase activity and melanin composition, Kumar's gingival pigmentation index and qualitative assessment of histological and immunohistochemical outcomes). Pain, itching, and discomfort score by any method or index (Visual Analog Score and Patient Satisfaction Surveys) Presurgical, surgical, and post‐surgical requirements and complications before and after depigmentation Adjuncts used with Vitamin C (laser, surgical blade, micro‐needling, etc.) Time required for the procedure Patient‐reported post‐operative outcomes

The results from each study and its characteristics of included studies are reported in Table 3 and a description of the factors is done as narrative synthesis below as follows:

1. Nature, type, mode, dosage, and frequency of application of vitamin C.
2. Efficacy of vitamin C in reducing gingival melanin pigmentation compared to other depigmentation procedures.
3. Effect of vitamin C on the gingival tissues.
4. Recurrence of pigmentation after vitamin C depigmentation compared to other conventional treatments.
5. The patient‐reported outcomes and the presence of any side effects.

**Table 3:** Characteristics of included studies.

Author/year/country of origin/study design	Age male/female ratio sample size	Smokers/non‐smokers comorbidities	Mode of application of Vitamin C	Study groups and sample size in each group	Outcome/parameters assessed follow‐up period	The index used for the assessment of gingival pigmentation	Study outcome	Limitations/adverse events
Shimada et al. (2009) Japan Prospective clinical split‐mouth trial	25–57 years (mean age: 37.2 years) 22 males; 51 females 73 subjects	38 smokers; 10 former smokers and 25 non‐smokers	Topical ascorbic acid‐glucoside gel (10%)	Test: Topical Vitamin C gel Control: Placebo gel	Changes in luminescence (L) In‐vitro assessment of inhibition of tyrosinase and melanin concentration 12 weeks	Spectro ‐photometric analysis (L)	The luminescence values changed significantly from 54.47 at baseline to 57.04 at 12 weeks in the test group (*p* < 0.05)	No direct assessment of changes in pigmentation indices. Short‐term follow‐up
Sheel et al. (2015) India Case report	18 years female patient	Non‐smoker	Ascorbic acid ampoules applied topically (10%/250 mg/mL)	Test group: Surgical scalpel along with topical applications of Vitamin C	Visual Analog Scores (VAS) Patient satisfaction using a three‐point scale.38 9 months	Dummet‐Gupta's Oral Pigmentation Index (DOPI) 39	Subjective decrease in pigmentation and absence of recurrence at 9 months. Reduction of VAS score from 1.8 intraoperatively to 1.5 10 days postoperatively. High patient satisfaction score of 3	Quantitative reduction in DOPI and recurrence rate not specified.
Yussif et al. (2017) Egypt Animal‐model study	15 adult male goats	not applicable	Intraepidermal injection (mesotherapy) using L‐ascorbic acid	Group I: Saline (5) Group II: 0.1 mL Vitamin C (5) Group III: 0.3 mL Vitamin C (5)	Immunohistochemical changes: Antibodies against human melanoma black (HMB‐45) and E‐Cadherin	—	Histological evidence revealed a marked reduction in melanin pigment and an increase in the number of cells with the perinuclear haloing in both groups II and III as compared to group I.	No quantitative assessment of histologic changes. No follow‐up.
Yussif et al. (2019) Egypt Prospective clinical parallel‐arm study	30 study participants Age > 18 years	Non‐smokers	Intraepidermal injection (mesotherapy) using L‐ascorbic acid	Test: Non‐surgical Vitamin C injections (15) Control: Scalpel depigmentation (15)	Changes in two pigmentation indices Visual Analog Score (VAS) for pain and itching. Patient satisfaction 9 months	Hanioka's Melanin Index Kumar's Gingival pigmentation index (GPI)	A significant difference in GPI in favor of the control group over the test group at 1 month. Significant reduction in VAS scores (from 5 at baseline to 0 on the 7th day) for itching and pain on the 2nd day (*p* = 0.0001), 3rd (*p* = 0.0001), and 7th day (*p* = 0.0015) when compared to scalpel depigmentation	Not reported
El‐Mofty et al. (2021) Egypt Prospective parallel‐arm clinical study	18–40 years with a mean age of 27.3 2 males and 8 females in both study groups 20 subjects	Non‐smokers	Intraepidermal injection (mesotherapy) using L‐ascorbic acid and Topical ascorbic acid‐glucoside gel (10%)	Group I: Vitamin C Mesotherapy (10) Group II: Topical Vitamin C (10)	Changes in DOPI satisfaction questionnaire modified from McGill Pain Questionnaire (Melzack 1975) Digital Melanin Area Fraction Analysis 6 months	DOPI	Median DOPI changed significantly from 2 at baseline to 1 at 6 months (*p* < 0.001) in Group I Median DOPI remained 1 at 6 months for Group II (*p* = 0.223) Both groups revealed a significant reduction in melanin area fraction at 6 months when compared to the baseline (*p* = 0.005 and *p* = 0.012, respectively)	Statistically significant differences between study groups at baseline may confound statistical analysis.
Mostafa et al. (2023) Egypt Case report	25 year female patient	Non‐smoker	1000 mg/mL ascorbic acid powder‐saline slurry applied after micro‐needling	—	Reduction of 2 pigmentation indices Qualitative assessment of pain, healing, and re‐pigmentation 6 months	DOPI and Hanioka's Melanin Index	Reduction of DOPI from 3 to 0 and Melanin Index score from 2 to 0. Light brown solitary re‐pigmentation at 6 months	Short‐term follow‐up with limited sample size. No histologic evaluation
Chaudhary et al. (2023) India prospective parallel‐arm clinical trial	18‐40 years 60% (18) were females and 40% (12) males 30 subjects	Exclusion of chronic smokers (criteria unspecified)	Intraepidermal injection (mesotherapy) using L‐ascorbic acid	Group I: Scalpel depigmentation (15) Group II: Vitamin C mesotherapy (15)	Changes in pigmentation and repigmentation assessed by graphical estimation VAS for pain Verbal scale for itching 3 months	DOPI	The non‐significant difference in reduction of pigmentation area at 1 month (32.27 with the control group and 37.07 with the test group, *p* = 0.932) Non‐significant influence on re‐pigmentation (*p* = 0.903) Significant reduction in VAS scores with the test group over the control group (0.73 and 3.33 respectively, *p* = 0.001)	Non‐quantitative classification of periodontal phenotypes Maxillary sextants are compared with mandibular sextants which may confound study results due to differences in area, phenotype, and intensity.
Dawar et al. (2022) India Case series	> 18 years 5 patients	Non‐smokers	Intraepidermal injection (mesotherapy) using L‐ascorbic acid	Intraepidermal injection (mesotherapy) using L‐ascorbic acid No control group	Change in gingival luminescence Pigmented surface area (PSA) Melanocyte count VAS score for pain Patient satisfaction survey 3 months	DOPI GPI	Significant reduction in the median GPI (*p* = 0.05), DOPI (*p* = 0.04), pigmented surface area (PSA) (*p* = 0.04), and (*p* = 0.04) at 1‐month follow‐up Between 1st and 3rd months, a marked improvement in the only change in gingival luminescence was noted (*p* = 0.04). Reduction in median melanocyte count from 102 to 52 at 3 months. Three out of five patients gave a high satisfaction score of 4 (scale of 0–4) and 2 gave a score of 3 at the 3‐month follow‐up. Median VAS pain score of 3 on the day of the procedure	No consideration of gingival biotype. Short‐term follow‐up.
Esmat et al. (2023) Randomized, parallel double‐blinded clinical trial Egypt	18 and 40 year 26 patients 5.4% males and 84.6% females	Non‐smokers	Intra‐mucosal injection of vitamin C (L‐ascorbic acid 1000 mg/5 mL)	Group 1: Intra‐mucosal injection of vitamin C (L‐ascorbic acid 1000 mg/5 mL) Group 2: Diode laser (980 nm, 1.5 W, continuous wave mode)	Clinical evaluation of pigmentation intensity and distribution was performed preoperatively and at 1, 2, and 3 months postoperatively. Dummett‐Gupta Oral Pigmentation Index (DOPI), and Gingival Pigmentation Index (GPI). Pain intensity and patients’ satisfaction were checked using VAS and questionnaires.	Dummett‐Gupta Oral Pigmentation Index (DOPI), and Gingival Pigmentation Index (GPI).	Pigmentation scores decreased significantly between pre‐operative visits and different follow‐up visits for both treatment modalities (*p* < 0.0001). When compared to the vitamin C mesotherapy group, the laser group demonstrated significantly lower gingival pigmentation scores (*p* < 0.0001). Both treatment modalities were equally satisfying for the patients.	Longer follow‐up periods are required to assess the recurrence rate.
Sandhu et al. (2023) Case series India	Two male patients aged 22 and 25 years	Non‐smokers	Topical application of Enshine Cream 15 g) along scalpel depigmentation	Case 1: Vitamin C + scalpel depigmentation Case 2: scalpel depigmentation	Dummett Oral Pigmentation Index (DOPI).	Dummett Oral Pigmentation Index (DOPI).	Improved healing and positive effects of vitamin C in the healing phase of gingival tissues following scalpel depigmentation	No quantitative data were reported for the DOPI score for both cases and no long‐term follow‐up.
Mostafa et al. (2023) Case series: prospective Riyadh, KSA	16 patients 17–35 years old; 8 women and 8 men	Non‐smokers	Ascorbic acid powder (1000 mg) and a Dermapen, (a pen‐like instrument with a handle and 12‐ 24 needles arranged in rows moving at a speed of 700 cycles/min).	Microneedling technique using the Dermapen with needles of 1.5 mm depth followed by topical Vitamin C powder (1000 mg/mL) mixed with saline was applied for 10 min and repeated after 2 weeks.	Hedin melanin index and Dummett Oral Pigmentation Index (DOPI) at baseline and follow up at one month	Hedin melanin index and Dummett Oral Pigmentation Index (DOPI)	Generally, healing was normal and satisfactory, and all patients achieved excellent aesthetic results with a reduction in both indices. Seven out of the 16 patients showed complete depigmentation of the gingiva, while nine patients displayed a reduction in their indices. The analysis using paired T‐tests demonstrated a statistically significant reduction in the post‐treatment DOPI score, with a mean difference of 1.8 ± 0.7 (95% CI: 0.17–1.49). Similarly, the HMI score was also lower post‐treatment, with a mean difference of 3.1 ± 0.7 (95% CI: 2.74–3.50).	The discomfort was experienced in most of the cases
Meenakshi and Subasree (2024) India Randomized clinical trial	16 participants Mandible sites Mean age: 26.7 ± 5.67 years. 50% (*n* = 8), and 50% women (*n* = 8).	Non‐smokers	Dermapen (Dr. Pen, Las Vegas, NV) device and topical ascorbic acid powder (1000 mg/mL) mixed with saline were applied over the gingiva for 10 min. three treatment sessions of microneedling, spaced out by 10 days	Case group: Microneedling with Vitamin C 1000 mg/mL) mixed with saline was applied for 10 min (three times) Control group: surgical depigmentation with a scalpel	Dummett‐Gupta Oral Pigmentation Index (DOPI) and early wound healing index by Marini et al. (2018) At baseline, one month, and three months follow up.	Dummett‐Gupta Oral Pigmentation Index (DOPI) and early wound healing index by Marini et al. (2018)	DOPI scores at baseline: 2.65 ± 0.16 and 2.61 ± 0.17 in the scalpel and microneedling with vitamin C. The mean DOPI score at the end of the third month was 1.67 ± 0.39 and 0.87 ± 0.17 in the surgical and MN with ascorbic acid groups, respectively. The healing index exhibited a statistically significant difference between the treated groups (*p* < 0.04) both at baseline and after 1 week. The healing index scores did not show a statistically significant difference within the same group in the surgical technique group at baseline and on the seventh day	Patients treated with conventional surgical techniques showed incomplete healing and ulceration on the first and seventh days after the procedure when compared to the microneedling technique with ascorbic acid.

The quantitative data in terms of mean and standard deviation, standard mean difference, gingival pigmentation score, pain score, and aesthetic score were noted. Qualitative data if present, about the procedure and pain score, were noted. The critical appraisal of included studies was assessed for their risk of bias using the Joanna Briggs Institute (JBI) scale. The risk of bias analysis was scored according to JBI risk of bias analysis and scored as follows: Studies with scores 0–4 were considered to have a high risk of bias; 5–7 were considered to have a moderate risk of bias and more than equal to 8 were considered to have a low risk of bias.

## 3 Results

### 3.1 Characteristics of the Individual Studies

**Figure 2:**
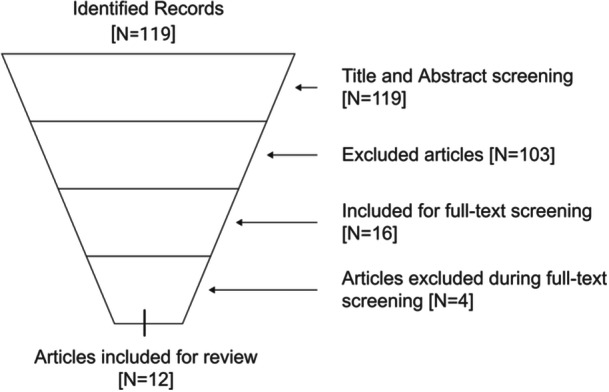
PRISMA flow diagram.

A total of 119 articles were obtained from all databases (Figure 2). After removing duplicates, 103 articles were eligible for titles and abstracts. Following title and abstract screening, 16 articles were included, out of which 4 articles were removed as they did not pertain to our particular focus question. Thus, a total of 12 studies were eligible for the review (Mostafa and Alotaibi 2022; Sheel et al. 2015; Shimada et al. 2009; Yussif et al. 2019, 2017; El‐Mofty et al. 2021; Chaudhary et al. 2023; Dawar et al. 2022; Mostafa et al. 2023; Meenakshi and Subasree 2024). Out of the 12 research articles, five were case reports/case series (Mostafa and Alotaibi 2022; Sheel et al. 2015; Dawar et al. 2022; Mostafa et al. 2023; Sandhu et al. 2023), one was an animal‐model study (goat model) (Yussif et al. 2017); five were clinical trials (Yussif et al. 2019; El‐Mofty et al. 2021; Chaudhary et al. 2023; Esmat et al. 2023; Meenakshi and Subasree 2024); one was a hybrid study with cell‐culture assessment as well as clinical evaluation on human (Shimada et al. 2009). Six studies were conducted in Egypt (Mostafa and Alotaibi 2022; Yussif et al. 2019, 2017; El‐Mofty et al. 2021; Esmat et al. 2023; Mostafa et al. 2023), five in India (Sheel et al.^2015^; Chaudhary et al. 2023; Dawar et al. 2022; Sandhu et al. 2023; Meenakshi and Subasree 2024), and one in Japan (Shimada et al. 2009). The sample size ranged from 20 to 73 subjects (Shimada et al. 2009; El‐Mofty et al. 2021). One study included both smokers and non‐smokers whereas four studies excluded smokers at the eligibility check (Shimada et al. 2009; Yussif et al.^2019^; El‐Mofty et al. 2021; Chaudhary et al. 2023; Dawar et al. 2022). The subjects included in two case‐report studies were self‐reported non‐smokers (Mostafa and Alotaibi 2022; Sheel et al. 2015). The mean age of the samples from all the studies ranged from 27.2 years to 37.2 years (Shimada et al. 2009; Yussif et al.^2019^) (Figure 2). The detailed characteristics of the demographic data from the included studies are also mentioned in Table 2. One case report showed high risk of bias (Sandhu et al. 2023); four studies depicted a moderate risk of bias (Mostafa and Alotaibi 2022; Sheel et al. 2015; El‐Mofty et al. 2021; Chaudhary et al. 2023); whereas seven studies had a low risk of bias (Shimada et al. 2009; Tricco et al. 2018; Yussif et al. 2019, 2017; Dawar et al. 2022; Esmat et al. 2023; Mostafa et al. 2023; Meenakshi and Subasree 2024) (Table S1–S3).

### 3.2 Type of Gingival Pigmentation and Method Used for Analysis

All studies carried out depigmentation for the removal of physiologic melanin pigments. The depigmentation was done for maxillary and mandibular anterior regions only (Mostafa and Alotaibi 2022; Sheel et al. 2015; Shimada et al. 2009; Yussif et al. 2019, 2017; El‐Mofty et al. 2021; Chaudhary et al. 2023; Dawar et al. 2022; Meenakshi and Subasree 2024). The classification and evaluation of changes in pigmentation were assessed using Dummet‐Gupta's Oral Pigmentation Index (DOPI) (Mostafa and Alotaibi 2022; Sheel et al. 2015; El‐Mofty et al. 2021; Dawar et al. 2022; Esmat et al. 2023; Sandhu et al. 2023; Mostafa et al. 2023; Meenakshi and Subasree 2024); Hanioka's melanin index (Mostafa et al. 2023; Mostafa and Alotaibi 2022; Yussif et al. 2019); Spectrophotometric analysis (Shimada et al. 2009; Dawar et al. 2022); inhibition of tyrosinase activity and melanin composition (Shimada et al. 2009); Kumar's gingival pigmentation index (Yussif et al. 2019; Dawar et al. 2022; Esmat et al. 2023); early wound healing score by Marini et al. (2018) (Meenakshi and Subasree 2024), and qualitative assessment of histological and immunohistochemical outcomes (Yussif et al. 2017; El‐Mofty et al. 2021). Five studies reported patient‐reported outcomes in terms of pain, discomfort, or itching using the visual analog scale (VAS) (Sheel et al. 2015; Yussif et al. 2019; Chaudhary et al. 2023; Dawar et al. 2022; Esmat et al. 2023); and the McGill pain and patient satisfaction questionnaire (El‐Mofty et al. 2021; Esmat et al. 2023).

### 3.3 Results of Individual Sources of Evidence and Synthesis of Results

#### 3.3.1 Mode, Dosage, Frequency of Application of Vitamin C

Studies tested the efficacy of vitamin C as an adjunct to scalpel depigmentation (Sheel et al. 2015) and as a stand‐alone therapy (Mostafa and Alotaibi 2022; Shimada et al. 2009; Yussif et al. 2019, 2017; El‐Mofty et al. 2021; Chaudhary et al. 2023; Dawar et al. 2022). Two studies compared the efficacy of vitamin C to scalpel depigmentation (Yussif et al. 2019; Chaudhary et al. 2023) and one study compared two modes of application of vitamin C (mesotherapy and topical) (El‐Mofty et al. 2021); two case series compared the role of vitamin C to scalpel (Sandhu et al. 2023); one RCT compared the role vitamin C to LASER (diode LASER) (Esmat et al. 2023). One animal study compared different concentrations of vitamin C as mesotherapy (Yussif et al. 2017). One clinical study compared the topical application of vitamin C to a placebo gel (Shimada et al. 2009). The mode of application of vitamin C included: topical application of ascorbic acid powder‐saline slurry (Mostafa and Alotaibi 2022; Mostafa et al. 2023); cotton roll‐assisted topical application of vitamin C capsule contents (Sheel et al. 2015); topical application of a vitamin‐C derivative gel composed of 10% ascorbic acid 2‐glucoside (Shimada et al. 2009; El‐Mofty et al. 2021) and intraepidermal injections (oral mesotherapy) of L‐ascorbic acid (Yussif et al. 2019, 2017; El‐Mofty et al. 2021; Chaudhary et al. 2023; Dawar et al. 2022; Esmat et al. 2023). L‐ascorbic acid and ascorbic acid 2‐glucosides were two derivatives of vitamin C that were employed for depigmentation procedures (Boo 2022; Yokota and Yahagi 2022).

Different doses of vitamin C (ascorbic acid) were used such as 1000 mg/mL (Mostafa and Alotaibi 2022; Mostafa et al. 2023); 10% ascorbic acid (Sheel et al. 2015); 10% ascorbic acid‐glucoside gel (Shimada et al. 2009) and 1–2 mL of 1 mg/5mL L‐ascorbic acid (ampoules) equivalent to 200–300 mg vitamin C concentration as 0.1 mL for each point with 2–3 mm apart, once a week for four to 5 weeks (Yussif et al. 2019, 2017; El‐Mofty et al. 2021; Chaudhary et al. 2023; Dawar et al. 2022). One study also compared the effect of 0.1 mL and 0.3 mL of vitamin C mesotherapy (Yussif et al. 2017). Another study applied vitamin C using an alternate approach and carried out micro‐needling for 30–40 s per tooth followed by a topical application for 10 min, twice at an interval of 2 weeks (Mostafa and Alotaibi 2022; Mostafa et al. 2023). When used as an adjunct to scalpel depigmentation, topical vitamin C was applied to surgically treated sites at weekly intervals for a month, followed by monthly intervals for 9 months (Sheel et al. 2015). In two prospective clinical trials topical gel application once daily after brushing at night, for 12 weeks was also tested (Shimada et al. 2009; El‐Mofty et al. 2021). The intraepidermal injections of vitamin C were done using different protocols. In three studies that carried out non‐surgical vitamin C mesotherapy, the agent was delivered using 30‐gauge syringes/insulin syringes either once a week for a maximum duration of 4 weeks (Yussif et al. 2017; Chaudhary et al. 2023; Dawar et al. 2022) or thrice at intervals of 1 week (El‐Mofty et al. 2021). Table 3 describes the characteristics of the included studies.

### 3.4 Efficacy of Vitamin C in Reducing Gingival Melanin Pigmentation Compared to Other Depigmentation Procedures

Vitamin C was found to be effective in reducing gingival pigmentation in all of the included studies. Mesotherapy (intra‐epithelial injection) was the most employed technique for vitamin C depigmentation (Yussif et al. 2019, 2017; El‐Mofty et al. 2021; Dawar et al. 2022). A study by El‐Mofty et al. (2021) compared the efficacy of mesotherapy versus topical depigmentation and found that intra‐epidermal injection has better reduction at 6 months (*p* < 0.001) (El‐Mofty et al. 2021). The DOPI value of two at baseline reduced to one at 6 months for epidermal injection; however, for topical application, the DOPI remained unchanged at 6 months (*p* = 0.223). Both groups revealed a significant reduction in melanin area fraction at 6 months when compared to the baseline (*p* = 0.005 and *p* = 0.012 respectively), with an insignificant inter‐group difference (*p* = 0.082). At 1 week and 6 months, there was a significant improvement in patient‐reported cosmetic changes for group I (*p* = 0.001 and *p* < 0.001, respectively). Shimada et al. evaluated the effect of the topical application of vitamin C compared to the placebo gel and found a significant change in luminescence value which changed from 54.47 at baseline to 57.04 at 12 weeks in the vitamin C group (*p* < 0.05). After 9 months, the pigmentation has decreased subjectively and there has been no recurrence. There was a decrease in VAS score from 1.8 intraoperatively to 1.5 at 10 days after surgery. In addition, a high patient satisfaction score of three was recorded (Shimada et al. 2009).

Another study by Yussif et al. compared vitamin C mesotherapy with scalpel depigmentation and revealed a significantly improved reduction in Kumar's Gingival pigmentation index for the scalpel group after 1 month (*p* = 0.003). At the end of 9 months, the results of the scalpel and vitamin C groups were comparable when measured using Hanioka's Melanin Index and Kumar's Gingival pigmentation. The itching sensation score (from 5 at baseline to 0 on the 7th day postoperatively) and pain score (on the 2nd, 3rd, and 7th day postoperatively) reduced significantly compared to scalpel depigmentation, which then became non‐significant at the 9th month (*p* = 0.46) (Yussif et al. 2019). Chaudhary et al. assessed the effect of vitamin C mesotherapy compared to scalpel and found no difference in the reduction of pigmentation area at 1 month (32.27 for the control group and for the vitamin C (37.07), (*p* = 0.932). No significant influence on repigmentation was noted (*p* = 0.903). A significantly favorable reduction in VAS scores with the vitamin C group over the control group was also observed (0.73 and 3.33 respectively, *p* = 0.001) (Chaudhary et al. 2023). A case series using oral vitamin C mesotherapy revealed a significant reduction in gingival pigmentation as evident by median gingival pigmentation index (*p* = 0.05), DOPI (*p* = 0.04), pigmented surface area (PSA) (*p* = 0.04), and change in luminescence (*p* = 0.04) at 1‐month follow up. When comparing 1‐ and 3‐month follow‐ups, only luminescence showed a marked improvement (*p* = 0.04). Another study showed that microneedling with adjunct topical administration of vitamin C resulted in excellent aesthetic outcomes after two applications at the end of 1 month. They observed that DOPI and melanin index scores of 3 and 2 respectively, reduced to 0 after two applications (Dawar et al. 2022). Topical application after scalpel depigmentation produced a significant improvement in DOPI (Sandhu et al. 2023). In 2023; Mostafa et al. (2023) also reported a significant reduction in melanin pigmentation upon the use of microneedling with topical application of vitamin C (twice application after 2 weeks). A statistically significant lower DOPI score post‐treatment (mean difference 1.8 ± 0.7, 95% confidence interval [CI]: 0.17–1.49, *p* ≤ 0.001) at the end of 1 month. Seven out of the 16 patients showed complete depigmentation of the gingiva, while nine patients displayed a reduction in their indices (Mostafa et al. 2023). A randomized clinical trial compared the efficacy of vitamin C mesotherapy to diode LASER and found that the pigmentation decreased significantly between pre‐operative visits and at 3‐months follow‐up visits for both the vitamin C and LASER group (*p* < 0.0001). However, the laser was found to have better results and lower gingival pigmentation scores compared to vitamin C at the follow‐up visits (*p* < 0.0001) (Esmat et al. 2023).

### 3.5 Effect of Vitamin C on the Gingival Tissues

As evidenced by in‐vitro and histopathological studies, vitamin C was found to induce biochemical and histological changes in the gingiva (Shimada et al. 2009; Yussif et al. 2017). A vitamin C derivative gel composed of 10% ascorbic acid 2‐glucoside resulted in a proportional, marked inhibition in the tyrosinase activity (*p* < 0.01), along with a significant decrease of 48% in the melanin composition (*p* < 0.05) in mouse melanoma cells when compared to placebo (Shimada et al. 2009). Similarly, another study on goats revealed a definite qualitative histologic reduction in melanin, causing perinuclear vacuolating and disruption of the intercellular contact between melanocytes and keratinocytes (Yussif et al. 2017). The study reported that the depigmenting effect was enhanced with a higher dose of vitamin C. The test groups also revealed a strong HMB‐45 antibody reaction with low levels of residual melanin granules concerning basal and suprabasal epithelial layers. Another study comparing vitamin C as mesotherapy, and topical form reported a significant reduction in histologic melanin area fraction at 6 months when compared to the baseline for both modalities (*p* = 0.005 and *p* = 0.012, respectively) (El‐Mofty et al. 2021). However, the inter‐group difference at 6 months was non‐significant. (*p* = 0.082). A case‐series study assessing melanocyte‐histopathologic count after vitamin C mesotherapy showed a reduction in median values from 102 at baseline to 52 at 3 months (Dawar et al. 2022).

### 3.6 Recurrence of Pigmentation After Vitamin C Depigmentation

The recurrence rate of pigmentation following vitamin C application was measured both quantitatively and qualitatively. Based on the quantitative data a recurrence of 32.59% was observed after 3 months of Vitamin‐C mesotherapy compared to 32.87% in the scalpel group (*p* = 0.903) (Chaudhary et al. 2023). Based on qualitative data occurrence of re‐pigmentation was noted (Mostafa and Alotaibi 2022; Sheel et al. 2015). Light brown solitary pigmented areas were observed at 6 months follow‐up with micro‐needling‐assisted vitamin C depigmentation (Mostafa and Alotaibi 2022). However, when used as a topical adjunct to scalpel depigmentation, no recurrence was observed up to a follow‐up of 9 months (Sheel et al. 2015). A prospective clinical trial using topical vitamin C gel revealed no recurrence at 12 weeks (Shimada et al. 2009). Intramucosal injections of vitamin C revealed no recurrence at 9 months which was comparable to scalpel depigmentation (Yussif et al. 2019). There are no studies comparing recurrence rates between different modalities of vitamin C or comparing vitamin C and cryotherapy‐assisted depigmentation.

### 3.7 Vitamin‐C Depigmentation and Patient‐Reported Outcomes

Five studies evaluated pain and satisfaction following vitamin C depigmentation, out of which four utilized the visual analog score (VAS) (Sheel et al. 2015; Yussif et al. 2019, 2017; Chaudhary et al. 2023; Dawar et al. 2022). Simultaneously, two studies quantitatively evaluated patient satisfaction (El‐Mofty et al. 2021; Dawar et al. 2022). The topical application of vitamin C on the scalpel depigmentation site exhibited a VAS score of 1.8 on the day of the procedure and 1.5 after 10 days (Sheel et al. 2015). vitamin C mesotherapy revealed a significant reduction in VAS scores for itching and pain compared to scalpel depigmentation, thus rendering it comparatively atraumatic (Yussif et al. 2019). Similarly, scalpel depigmentation depicted a significantly higher mean VAS score when compared to vitamin C mesotherapy after 24 h of the procedure (*p* = 0.001) (Chaudhary et al. 2023). A case series of five patients who received vitamin C mesotherapy revealed a median pain score of three on the day of the procedure (Dawar et al. 2022). The study comparing vitamin C mesotherapy and topical application reported that 100% of the patients in both study groups experienced no pain on the day of the surgery, as well as 1 week after the procedure (El‐Mofty et al. 2021). The study evaluating the microneedling approach only mentioned a qualitative reduction in pain and discomfort after the third postoperative day (Mostafa and Alotaibi 2022). In terms of patient satisfaction, a comparison between mesotherapy and topical vitamin C showed significant differences in favor of the former in terms of patient‐reported cosmetic change at 1 week and 6 months (*p* = 0.001 and *p* < 0.001, respectively) (El‐Mofty et al. 2021); 100% of the patients in the topical application group were unsatisfied with the treatment outcome compared to 40% in the mesotherapy group (*p* = 0.011) (El‐Mofty et al. 2021). In the case series using vitamin C mesotherapy, three out of five patients gave a high satisfaction score of four, and two patients gave a score of three at the 3‐month follow‐up (Dawar et al. 2022). Multiple studies have corroborated that pain, itching, and discomfort are prominently reduced using itamin C, regardless of the mode of application (Yussif et al. 2019; El‐Mofty et al. 2021; Chaudhary et al. 2023; Dawar et al. 2022). Even intraepidermal injections of vitamin C have shown excellent patient acceptance when contrasted with scalpel depigmentation (Yussif et al. 2019; Chaudhary et al. 2023). Esmat et al. (2023) compared the patient satisfaction score and pain score using a questionnaire and VAS between patients treated with LASER and vitamin C mesotherapy for depigmentation. The authors found A statistically significant difference (*p* = 0.005) in immediate postoperative pain scores between the two groups, favoring the LASER group. However, no significant differences in pain scores on the 1st and 7th postoperative days were detected between the two groups. No difference was seen in the patient's satisfaction and acceptance of the treatment method between the two groups as assessed by the questionnaire for assessing the aesthetic/cosmetic appearance after 3 months, fulfillment of expectation, and willingness to repeat the procedure (Esmat et al. 2023).

## 4 Discussion and Summary of Evidence

The aim of this review is to provide an evaluation of the effectiveness of vitamin C in the management of gingival pigmentation. Vitamin C is known for its anti‐inflammatory, antioxidant, and wound‐healing properties, which have led to its common use in dermatology and cosmetics for depigmentation and skin lightening. It is also being considered for use in the management of gingival hyperpigmentation. It is noted that studies have compared both topical and mesotherapy for vitamin C application to reduce the melanin pigments in the gingiva. It has been observed that microneedling with a dermapen can create pinpoint holes in the gingival epithelium, with a depth of up to 0.2–1 mm. Subsequently, vitamin C is applied to the affected area and left for approximately 10–20 min, during which the vitamin C is permitted to enter the epithelium. Vitamin C can be injected into the gingiva via microneedles or applied directly, with the latter being less effective. It can be used as an atraumatic method of reducing heavy physiologic melanin deposits in the gingiva. It would seem that the most effective results in reducing clinical pigmentation can be achieved by repeating the application of vitamin C, either via intradermal injection or micro‐needling, with a gap of 10–15 days between each application. Studies have indicated that vitamin C may be an effective approach to reducing gingival pigmentation, and it may offer a less invasive alternative to other methods such as scalpel, bur, and electrocautery (Hanioka et al. 2005; Miot et al. 2012). It is thought that the use of vitamin C with microneedling may help to improve patient acceptance and ease of procedure compared to surgical de‐epithelization. Additionally, the anti‐inflammatory and antioxidant properties of vitamin C may help to accelerate wound healing and reduce post‐operative inflammation and pain following de‐epithelization of gingiva with a scalpel/bur.

Vitamin C depigmentation is an effective treatment for young adults with special needs, including those with mental retardation and developmental anomalies. It is important to note, however, that a mesotherapy technique involving multiple injections may be unacceptable and uncomfortable for some patients, particularly those with a needle phobia. Furthermore, the necessity for multiple visits for vitamin C mesotherapy presents a significant challenge in terms of patient compliance.

However, we must highlight some of the limitations of the current evidence. There is insufficient evidence that quantitatively evaluates the recurrence rate and efficacy of vitamin C depigmentation in the long term. It is crucial to acknowledge that one study evaluated the impact of vitamin C on smokers, which could potentially bias the results due to the detrimental effects of smoking on gingival tissues. Future studies must therefore compare the effect of re‐pigmentation following vitamin C application in smokers versus non‐smokers. We could find no studies comparing the role of vitamin C with laser depigmentation. Furthermore, it is essential to determine whether vitamin C can be used as a monotherapy or if it should always be used in conjunction with scalpels or lasers, as no studies have done so. The limited number of studies that evaluated the effect of topical vitamin C preparations demonstrated unequivocally that a reduction in oral pigmentation indices was observed even after topical application for 1 week (Shimada et al. 2009; El‐Mofty et al. 2021). One case report evaluated the effect of topical vitamin C as an adjunct to scalpel depigmentation and found a reduction in pigmentation that did not recur over a 9‐month follow‐up period (Sheel et al. 2015). A clinical trial is required to validate the suggestion that its efficacy will extend over a longer period, given the absence of a control group and the fact that the results were observed only in one patient (Sheel et al. 2015). Furthermore, no studies have considered the biotype/phenotype of the gingiva when assessing the role of vitamin C as an adjunct to gingival depigmentation. The maxillary gingiva is thicker than the mandibular region, so the depth of penetration of vitamin C will vary among patients. The gingiva ranges from 0.2 mm to 3 mm in thickness/phenotype. Furthermore, a quantitative assessment of wound healing following vitamin C depigmentation, along with a comparison with existing treatment modalities, must be conducted. Furthermore, the application of vitamin C to treat pathologic gingival pigmentation represents another potential research area. However, future studies should assess the long‐term effect of using different modes of applications of vitamin C for gingival depigmentation and compare its effectiveness and recurrence rate compared to other depigmentation procedures.

## 5 Conclusion

Vitamin C application either topically or via microneedling is a good treatment option for managing gingival pigmentation, with mesotherapy (intra‐epithelial injection) being the most effective outcome. Vitamin C application for gingival pigmentation is a less invasive alternative to other methods such as scalpel, bur, and electrocautery. Vitamin C with micro‐needling has better patient acceptance and ease of procedure compared to scalpel de‐epithelization. Vitamin C has shown good anti‐inflammatory and antioxidant properties that help accelerate wound healing and reduce post‐operative inflammation and pain.

## Author Contributions

Conceptualization: Shravya Marcherla, Malvika Shyamkumar, and Aditi Chopra. Methodology: Shravya Marcherla, Malvika Shyamkumar, Shubhankar Mehrotra, and Aditi Chopra. Development or design of methodology; Creation of models; Software: Malvika Shyamkumar and Aditi Chopra. Validation: Malvika Shyamkumar, Shubhankar Mehrotra, Aditi Chopra, and Marwa Madi. Formal analysis: Malvika Shyamkumar, Shubhankar Mehrotra, and Aditi Chopra. Investigation: Malvika Shyamkumar, Aditi Chopra, Shubhankar Mehrotra, and Shravya Marcherla. Resources: Malvika Shyamkumar, Aditi Chopra, and Shubhankar Mehrotra. Data curation: Malvika Shyamkumar, Aditi Chopra, Shubhankar Mehrotra, and Shravya Marcherla. Writing – original draft: Malvika Shyamkumar, Aditi Chopra, Shubhankar Mehrotra, Shravya Marcherla, and Marwa Madi. Writing – review and editing: Malvika Shyamkumar, Aditi Chopra, Shubhankar Mehrorta, and Shravya Marcherla. Visualization: Aditi Chopra. Supervision: Aditi Chopra. Project administration: Aditi Chopra. Funding acquisition: Aditi Chopra. All authors approved the final version to be published.

## Funding

The authors received no specific funding for this work.

## Ethics Statement

The authors have nothing to report.

## Consent

The authors have nothing to report.

## Conflicts of Interest

The authors declare no conflicts of interest.

## Supporting information


**Supplementary Table 1:** Risk of bias analysis using JBI tools for randomized clinical trial.

**Supplementary Table 2:** Risk of bias analysis using JBI tools for case report.

**Supplementary Table 3:** Risk of bias analysis using JBI tools for case series

## Data Availability

Data will be available upon request via email to the corresponding author.
