# Supplementary material for: How Effective Is Vitamin C for Gingival Depigmentation? A Scoping Review
Source: Clin Exp Dent Res. 2026 Mar 11;12(2):e70272. doi: 10.1002/cre2.70272 (PMC12976973; doi:10.1002/cre2.70272)
Supplement: Supplementary file 1 — Supplementary Table 1: Risk of bias analysis using JBI tools for randomized clinical trial. [file CRE2-12-e70272-s001.docx]

| Supplementary Table 1: Risk of bias analysis using JBI tools for randomized clinical trial | | | | | | | | | | | | | | | | | | | | | | | |
| --- | --- | --- | --- | --- | --- | --- | --- | --- | --- | --- | --- | --- | --- | --- | --- | --- | --- | --- | --- | --- | --- | --- | --- |
| **Author** | **Shimada et al. (2009)** | | | **El-Mofty et al. (2021)** | | | **Chaudhary et al. (2022)** | | | **Esmat et al. 2023** | | | **Yusiff et al. 2017** | | | **Yusiff et al. 2019** | | | **Meenakshi and Subasree, 2024** | | | | |
| Criteria | Yes | No | Unclear | Yes | No | Unclear | Yes | No | Unclear | Yes | No | Unclear | Yes | No | Unclear | Yes | No | Unclear | Yes | No | | Unclear | |
| 1.        Was true randomization used for the assignment of participants to treatment groups? | Yes |  |  | Yes |  |  | Yes |  |  | Yes |  |  | Yes |  |  | Yes |  |  | Yes |  | |  | |
| 2.        Was allocation to treatment groups concealed? |  | No |  |  | No |  |  |  | Unclear |  | No |  |  | No |  |  | No |  |  | No | |  | |
| 3.        Were treatment groups similar at the baseline? |  |  | Unclear |  | No |  |  | No |  | Yes |  |  | Yes |  |  | Yes |  |  | Yes |  | |  | |
| 4.        Were participants blind to treatment assignment? | Yes |  |  |  | No |  |  | No |  |  | No |  |  | No |  |  | No |  |  | No | |  | |
| 5.        Were those delivering treatment blind to treatment assignment? | Yes |  |  |  | No |  |  | No |  |  | No |  |  | No |  |  | No |  |  | No | |  | |
| 6.        Were outcomes assessors blind to treatment assignment? |  |  | Unclear |  |  | Yes |  |  | Unclear |  |  | Unclear |  |  | Unclear |  |  | Unclear |  |  | | Unclear | |
| 7.        Were treatment groups treated identically other than the intervention of interest? | Yes |  |  | Yes |  |  | Yes |  |  | Yes |  |  | Yes |  |  | Yes |  |  | Yes |  | |  | |
| 8.        Was follow up complete and if not, were differences between groups in terms of their follow up adequately described and analyzed? | Yes |  |  | Yes |  |  | Yes |  |  | Yes |  |  | Yes |  |  | Yes |  |  | Yes |  |  | | |
| 9.        Were participants analyzed in the groups to which they were randomized? | Yes |  |  | Yes |  |  | Yes |  |  | Yes |  |  | Yes |  |  | Yes |  |  | Yes |  |  | | |
| 10.     Were outcomes measured in the same way for treatment groups? | Yes |  |  | Yes |  |  | Yes |  |  | Yes |  |  | Yes |  |  |  |  |  | Yes |  |  | | |
| 11.     Were outcomes measured in a reliable way? | Yes |  |  |  | No |  | Yes |  |  | Yes |  |  | Yes |  |  |  |  |  | Yes |  |  | | |
| 12.     Was appropriate statistical analysis used? | Yes |  |  | Yes |  |  | Yes |  |  | Yes |  |  | Yes |  |  | Yes |  |  | Yes |  | | |  |
| 13.     Was the trial design appropriate, and any deviations from the standard RCT design (individual randomization, parallel groups) accounted for in the conduct and analysis of the trial? | Yes |  |  |  | No |  |  |  | Unclear | Yes |  |  | Yes |  |  | Yes |  |  |  |  | | | No |
| **TOTAL NO. of YES** | **9 (low-risk of bias)** | | | **6 (moderate risk of bias)** | | | **7 (moderate risk of bias)** | | | **8 (low risk of bias)** | | | **8 (low risk of bias)** | | | **7 (low risk of bias)** | | | **8 (low risk of Bias)** | | | | |
