# Supplementary material for: How Effective Is Vitamin C for Gingival Depigmentation? A Scoping Review
Source: Clin Exp Dent Res. 2026 Mar 11;12(2):e70272. doi: 10.1002/cre2.70272 (PMC12976973; doi:10.1002/cre2.70272)
Supplement: Supplementary file 2 — Supplementary Table 2: Risk of bias analysis using JBI tools for case report. [file CRE2-12-e70272-s003.docx]

| **Table 2: Risk of bias analysis of the study for case report** | | | | | | |
| --- | --- | --- | --- | --- | --- | --- |
| **AUTHOR** | **Sheel et al. (2015)** | |  | **Mostafa et al. (2022)** | | |
| CRITERIA | YES | NO | UNCLEAR | YES | NO | UNCLEAR |
| 1. Were patient’s demographic characteristics clearly described? | Yes |  |  | Yes |  |  |
| 2. Was the patient’s history clearly described and presented as a timeline? | Yes |  |  | Yes |  |  |
| 3.  Was the current clinical condition of the patient on presentation clearly described? | Yes |  |  | Yes |  |  |
| 4. Were diagnostic tests or assessment methods and the results clearly described? | Yes |  |  | Yes |  |  |
| 5. Was the intervention(s) or treatment procedure(s) clearly described? | Yes |  |  | Yes |  |  |
| 6. Was the post-intervention clinical condition clearly described? |  | No |  |  | No |  |
| 7. Were adverse events (harms) or unanticipated events identified and described? |  | No |  |  | No |  |
| 8. Does the case report provide takeaway lessons? | Yes |  |  | Yes |  |  |
| **TOTAL NO OF YES** | **7**  **(moderate-risk of bias)** |  |  | **7 (moderate-risk of bias)** |  |  |
