# Supplementary material for: How Effective Is Vitamin C for Gingival Depigmentation? A Scoping Review
Source: Clin Exp Dent Res. 2026 Mar 11;12(2):e70272. doi: 10.1002/cre2.70272 (PMC12976973; doi:10.1002/cre2.70272)
Supplement: Supplementary file 3 — Supplementary Table 3: Risk of bias analysis using JBI tools for case series [file CRE2-12-e70272-s002.docx]

| **Table 3: Risk of bias analysis of the study for case series** | | | | | | | | | |
| --- | --- | --- | --- | --- | --- | --- | --- | --- | --- |
| **AUTHOR** | **Dawar et al. (2022)** | | | **Sandhu et al. (2023)** | | | **Mostafa et al. (2023)** | | |
| CRITERIA | YES | NO | UNCLEAR | YES | NO | UNCLEAR | YES | NO | UNCLEAR |
| 1. Were there clear criteria for inclusion in the case series? | Yes |  |  | Yes |  |  | Yes |  |  |
| 2. Was the condition measured in a standard, reliable way for all participants included in the case series? | Yes |  |  | Yes |  |  | Yes |  |  |
| 3. Were valid methods used for the identification of the condition for all participants included in the case series? | Yes |  |  | Yes |  |  | Yes |  |  |
| 4. Did the case series have consecutive inclusion of participants? | Yes |  |  | Yes |  |  | Yes |  |  |
| 5. Did the case series have complete inclusion of participants? | Yes |  |  |  | No |  | Yes |  |  |
| 6. Was there clear reporting of the demographics of the participants in the study? |  | No |  |  | No |  | Yes |  |  |
| 7. Was there clear reporting of clinical information of the participants? | Yes |  |  |  | No |  | No |  |  |
| 8. Were the outcomes or follow-up results of cases clearly reported? | Yes |  |  |  | No |  | Yes |  |  |
| 9. Was there clear reporting of the presenting site(s)/clinic(s) demographic information? |  | No |  |  | No |  | Yes |  |  |
| 10. Was statistical analysis appropriate? |  | No |  |  | No |  |  | No |  |
| **TOTAL NO. OF YES** | **7 (low-risk of bias)** |  |  | **4 (High-risk of bias)** |  |  |  | **8 (low risk of bias)** |  |
